# Supplementary material for: Exposure to Ambient Air Pollution and the Incidence of Dementia in the Elderly of England: The ELSA Cohort
Source: Int J Environ Res Public Health. 2022 Nov 29;19(23):15889. doi: 10.3390/ijerph192315889 (PMC9736331; doi:10.3390/ijerph192315889)
Supplement: Supplementary file 1 [file ijerph-19-15889-s001.zip › ijerph-1983951-supplementary.pdf]

Supplementary Material

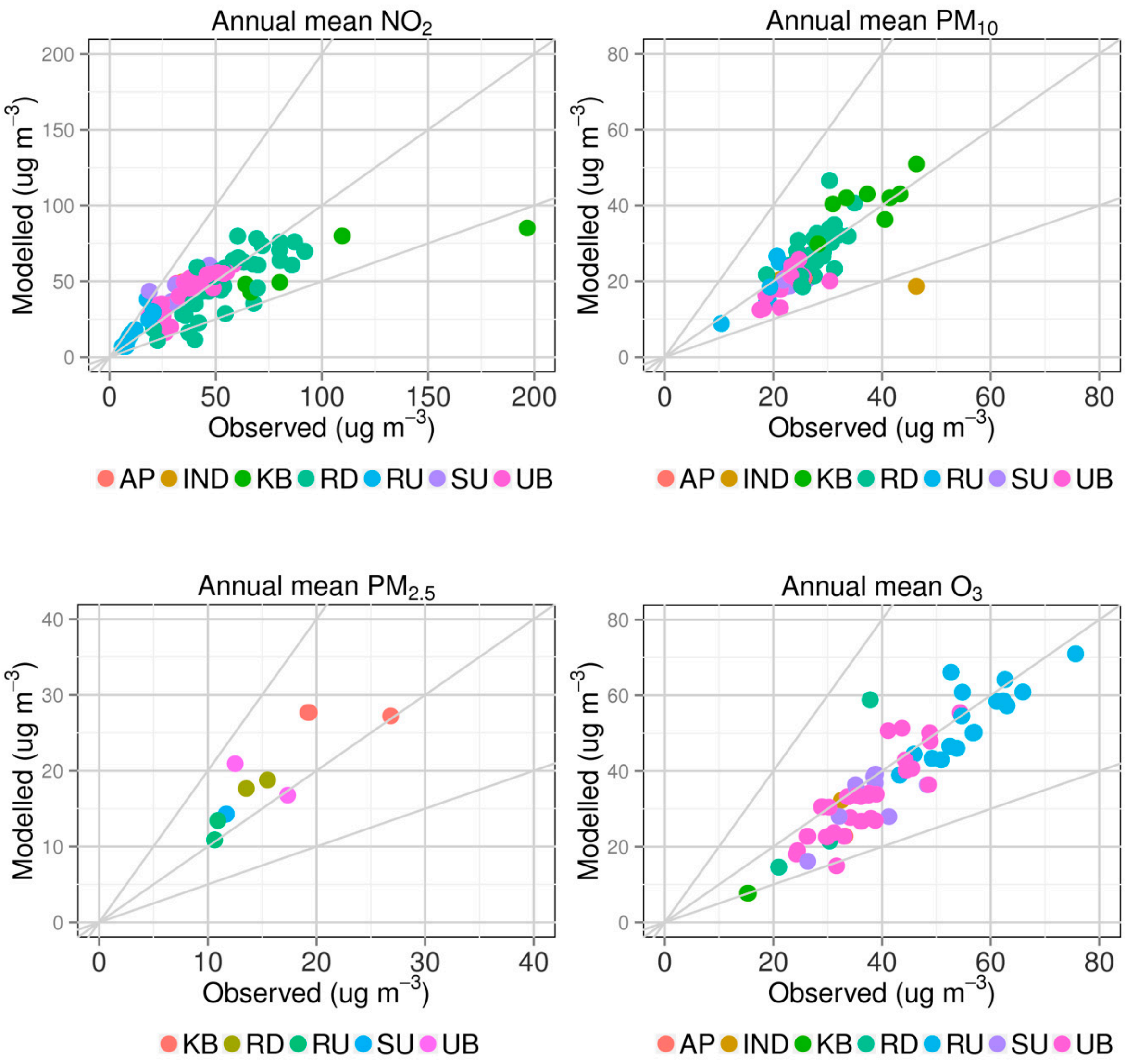

Figure S1. CMAQ-urban 2004 model validation. Observed (monitor concentrations) plotted against modelled concentrations. Monitor site type denoted by colour. **AP**: airport; **IND**: industrial; **KB**: kerbside; **RD**: roadside; **RU**: rural; **SU**: suburban; **UB**: urban background.

Table S1. CMAQ-urban 2004 model performance and validation in comparison to measured concentrations of NO_2, PM_{10}, $PM_{2.5}$ and ozone.

Pollutant	N Monitor Sites	RMSE	r
**NO_2**	122	15.32	0.78
**PM_{10}**	88	5.13	0.78
**$PM_{2.5}$**	10	5.04	0.82
**Ozone**	69	7.11	0.91

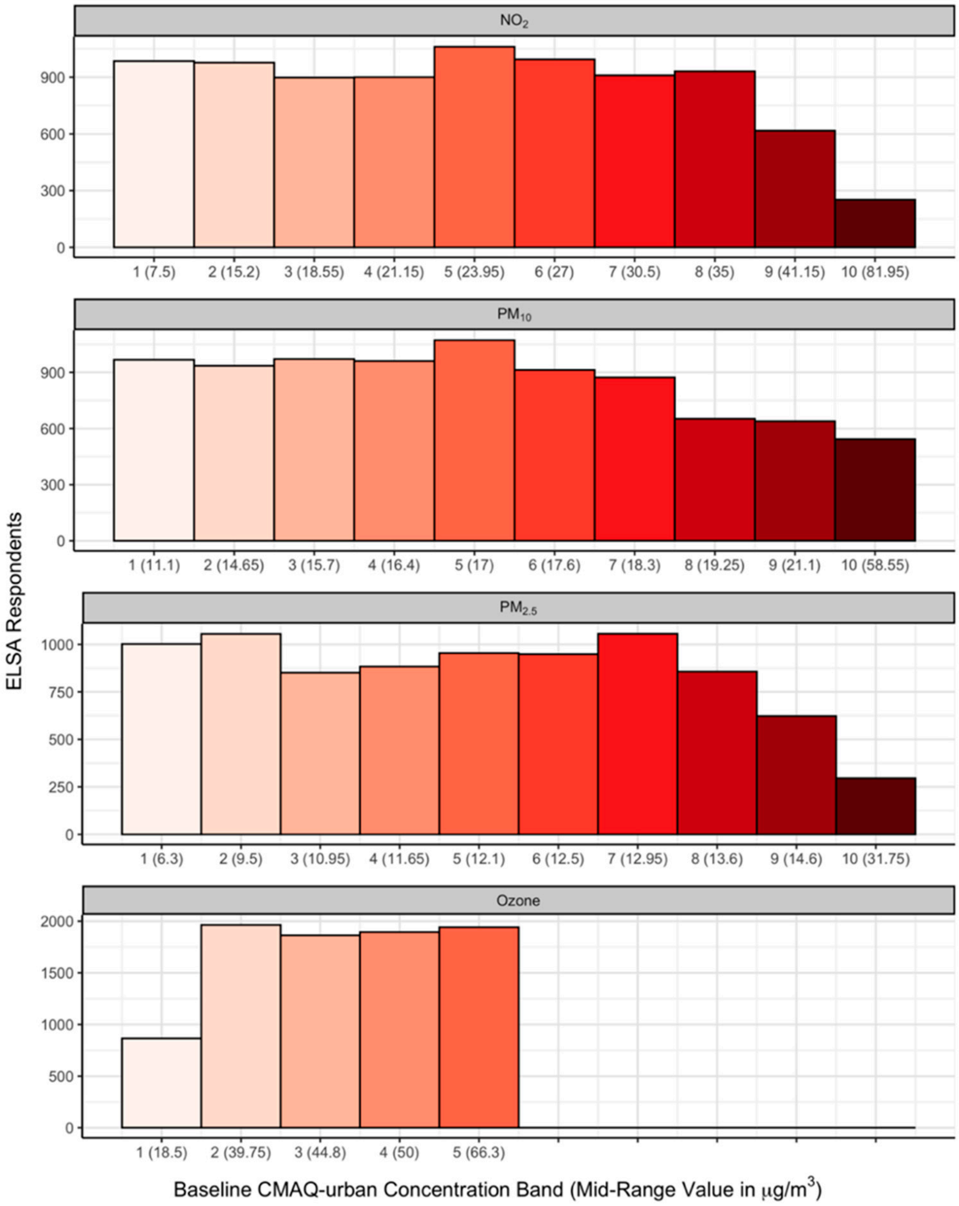

Figure S2. Distribution of ELSA respondents included in analysis of dementia incidence linked to CMAQ-urban exposure percentiles at baseline; 2004 concentration estimates (μg/m^3 mid-range values of each percentile range provided).

Table S2. Spearman correlation coefficients between assigned baseline CMAQ-urban modelled pollutant concentrations to ELSA respondents.

	**Spearman Correlation Coefficient**			
	**NO_2**	**PM_{10}**	**$PM_{2.5}$**	**Ozone**
**NO_2**		0.49	0.83	−0.9
**PM_{10}**	0.49		0.71	−0.41
**$PM_{2.5}$**	0.83	0.71		−0.8
**Ozone**	−0.9	−0.41	−0.8	

Table S3. Mean baseline CMAQ-urban modelled pollutant concentrations assigned to ELSA respondents by 10-year baseline age band.

	Mean Baseline Concentration (μg/m^3)			
**Baseline Age Band**	**NO_2**	**PM_{10}**	**$PM_{2.5}$**	**Ozone**
50–59	25.8	18.8	12.2	46.5
60–69	25.5	19.4	12.2	46.8
70–79	25.2	19.2	11.9	47.5
80–89	25.1	19.6	12	47.3
90+	23.5	19	12.1	48.6

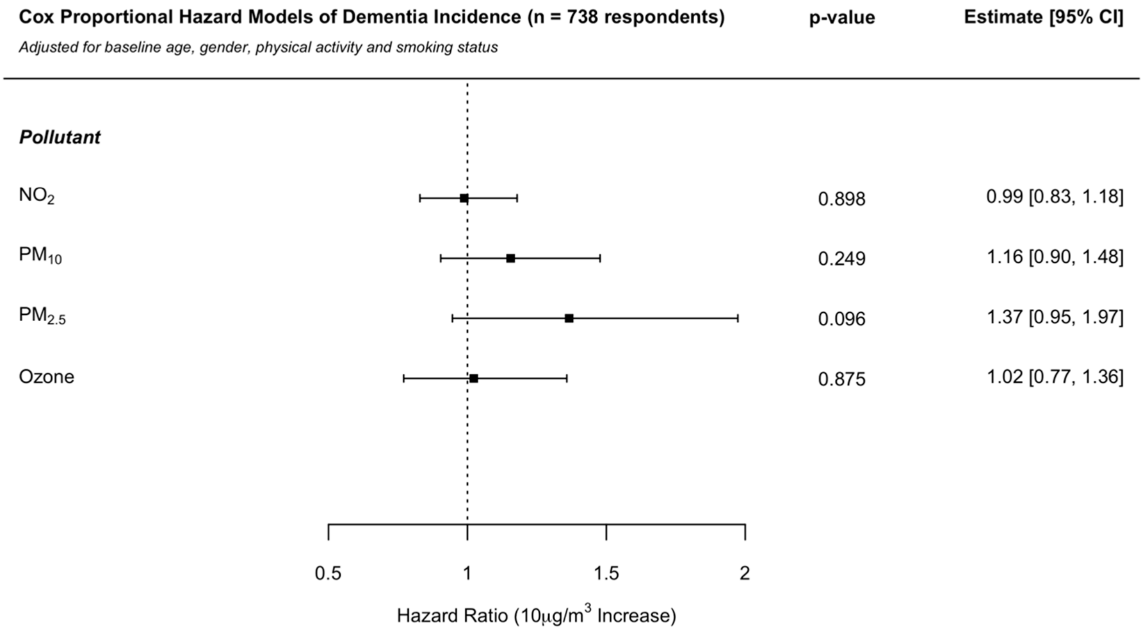

Figure S3. Cox proportional hazards model hazard ratios per 10μg/m^3 increase in NO_2, PM_{10}, $PM_{2.5}$ and ozone concentrations on dementia incidence in London-dwelling ELSA respondents (n = 738). Independent single pollutant models adjusted for baseline age, gender, physical activity and smoking status.

Table S4. Dementia cases reported in published studies assessing the incidence of dementia in ELSA respondents. Baseline ELSA wave utilised in the study, the number of follow-up waves included and dementia assignment method also provided. **SR**: self-reported; **IQCODE**: assignment via proxy interview; **HES**: hospital episode statistics linkage; **PM**: prescribed medicine use.

Published Article	ELSA Respondents	Dementia Cases	% of Sample	Baseline Wave	Follow-Up Waves	Dementia Assignment
Fancourt et al. (2020)	9550	893	9.35	2	6	SR, IQCODE
Cadar et al. (2018)	6220	463	7.44	1	6	SR, IQCODE
Hackett et al. (2018)	3932	289	7.35	1	6	SR, IQCODE
Ma et al. (2020)	6582	453	6.88	1	7	SR, IOCODE, HES
Feter et al. (2021)	9275	631	6.80	1	7	SR, IQCODE
Fancourt et al. (2018)	3911	246	6.29	2	5	SR, IQCODE
Almeida-Meza et al. (2020)	12,280	602	4.90	1	7	SR, IOCODE
Kontari et al. (2019)	4859	216	4.45	2	5	SR, IQCODE
Rogers et al. (2017)	8722	365	4.18	1	5	SR
d'Orsi et al. (2017)	8238	301	3.65	1	5	SR, IQCODE
Hackett et al. (2019)	4171	142	3.40	2	5	SR, IQCODE
Khondoker et al. (2017)	10,055	340	3.38	1	5	SR, IQCODE
Soni et al. (2019)	11,391	378	3.32	1	5	SR, IQCODE
Rafnsson et al. (2020)	6677	220	3.29	2	4	SR, IQCODE
Dregan et al. (2013a)	10,809	352	3.26	1	4	SR
Davies-Kershaw et al. (2018)	8648	275	3.18	2	5	SR, IQCODE, PM
Davies-Kershaw et al. (2017)	8780	269	3.06	2	5	SR, IQCODE, PM
Deckers et al. (2019)	6346	192	3.03	3	4	SR, IQCODE
